# Developing a Quantifying Device for Soft Tissue Material Properties around Lumbar Spines

**DOI:** 10.3390/bios11030067

**Published:** 2021-02-28

**Authors:** Song Joo Lee, Yong-Eun Cho, Kyung-Hyun Kim, Deukhee Lee

**Affiliations:** 1 Center for Bionics, Biomedical Research Institute, Korea Institute of Science and Technology, Seoul 02792, Korea; 2 Division of Bio-Medical Science & Technology, Korea Institute of Science and Technolgy (KIST) School, Korea University of Science and Technology, Seoul 02792, Korea; 3 Department of Neurosurgery, Spine and Spinal Cord Institute, Gangnam Severance Spine Hospital, Yonsei University College of Medicine, Seoul 02792, Korea; yecho@yuhs.ac (Y.-E.C.); nskhk@yuhs.ac (K.-H.K.); 4 Center for Healthcare Robotics, AI and Robot Institute, Korea Institute of Science and Technology, Seoul 02792, Korea

**Keywords:** quantifying device, soft tissue material properties, lumbar

## Abstract

Knowing the material properties of the musculoskeletal soft tissue could be important to develop rehabilitation therapy and surgical procedures. However, there is a lack of devices and information on the viscoelastic properties of soft tissues around the lumbar spine. The goal of this study was to develop a portable quantifying device for providing strain and stress curves of muscles and ligaments around the lumbar spine at various stretching speeds. Each sample was conditioned and applied for 20 repeatable cyclic 5 mm stretch-and-relax trials in the direction and perpendicular direction of the fiber at 2, 3 and 5 mm/s. Our device successfully provided the stress and strain curve of the samples and our results showed that there were significant effects of speed on the young’s modulus of the samples (*p* < 0.05). Compared to the expensive commercial device, our lower-cost device provided comparable stress and strain curves of the sample. Based on our device and findings, various sizes of samples can be measured and viscoelastic properties of the soft tissues can be obtained. Our portable device and approach can help to investigate young’s modulus of musculoskeletal soft tissues conveniently, and can be a basis for developing a material testing device in a surgical room or various lab environments.

## 1. Introduction

The human musculoskeletal soft tissue has viscoelastic properties showing time and history-dependent behaviors [1,2]. The behaviors can occur because skeletal muscle, ligament, and tendon are formed by a complex hierarchical structure, consisting of about 80% water, 3% fat, and 10% collagenous fibers, and interact with each other [2]. Knowing the material properties of the musculoskeletal soft tissue could be important to develop rehabilitation therapy and surgical procedures.

Especially, due to recent advances in medical technology, image-guided surgeries are widely performed by using computerized-tomography (CT), magnetic resonance imaging (MRI), fluoroscopic, or X-ray images [3]. Like cerebral nerve surgery or implant surgery, these image-guided surgical procedures are performed when a surgeon is not directly able to see the affected part of the patient or when major nerves and organs in the patients’ body are avoided [3,4,5]. In the case of performing the image-guided surgery, registration processes are performed to match the coordinates of the scanned images of a patient and the actual body part coordinates of the patient [3,4,6,7,8]. The operation can be performed in real-time while watching the pre-recorded scanned images on the monitor. During the image-guided surgery, over-incision, and a large amount of X-ray exposure can cause significant side effects to patients. Thereby, advanced image registration technologies are needed to minimize incision sizes and reduce amounts of X-ray exposure, especially for spine surgery [9]. 

To develop advanced image registration technologies, a dynamic biomechanical model might be needed to explain the mechanical principles of body organs and tissues. In the biomechanical model, anatomical shape and mechanical and material properties of human hard and soft tissues are needed. Generally, a tensile testing device is used to determine the material properties of the tested material. By pulling the material in the opposite direction at a constant speed, material properties such as yield strength, tensile strength, elongation, and elastic modulus can be measured. Commercially available tensile testing devices such as Instron can measure the material properties of soft tissue. However, this device may not be practical to use in a surgical room as it requires a large room to set up and it is difficult to move around. A soft tissue elastomer, which is small and portable, has been developed to measure Young’s modulus of soft tissue with the indentation of the tissue [10]. Thereby, there is a limitation of measuring the anisotropic, nonlinear, and viscoelastic properties of soft tissues [10].

Thus, the purpose of this study was to develop a portable quantifying device for anisotropic and viscoelastic material properties of musculoskeletal soft tissue that has the following capabilities: (1) controlling speeds of the quantifying device, (2) allowing a different initial length setup, and (3) providing a strain and stress curve and young’s modulus of the materials. The feasibility of the quantifying device was demonstrated by comparing the properties measured using the quantifying device and Instron and lumbar musculoskeletal soft tissue specimens from a cadaver.

## 2. Materials and Methods

### 2.1. A quantifying Device for Soft Tissue Material Properties

**Figure 1:**
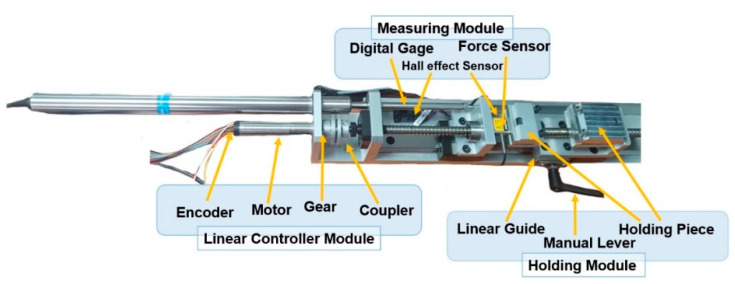
Quantifying device (QD) consisting of a linear controller module, holding module, and measuring module.

The quantifying device (QD) was developed to quantify soft tissue material properties (Figure 1). The QD consisted of a holding module, linear controller module, and measuring module. The holding module held the tissue sample by clamping each end of the sample. Because the initial length of the sample can be different, a manual lever on a linear guide was added to easily fix the length of the sample without any extra power or controlling the motor. Various thicknesses of samples could be positioned between two brackets and there were two screws on the top piece that could adjust the holding thickness so that the sample can be fixed between the brackets without structural damages to the test sample. 

The linear controller module consisted of a brushless DC servomotor with an EPOS positioning controller (Maxon, Sachseln, Switzerland) and included an encoder with a harmonic gear (a reduction ratio of 83:1). The rotation was transmitted through a coupler to move a linear guide. The positioning controller also included two Hall effect sensors locating at the bottom of the system unit so that they could function as a limit switch for a safety mechanism. The controller communicated with the computer via a USB connection. 

The measuring module consisted of a force sensor (Futek, LSB200, Irvine, CA, USA) to measure the tensile force (up to 445 N) and a digital gauge (Magnescale DK100PR5, Tokyo, Japan) to measure the elongation of the specimen (up to 100 mm with 4 μm accuracy). Those specs were sufficient to quantify our study since the elongation was in the order of mm, and the force was in the order of N. The force sensor was located between the bracket and the side holder where the linear motor with a guide moved (Figure 1). In this setup, when the initial length of the specimen was changed with the manual maneuver during multiple test trials, accurately tracking the linear position of the specimen could be difficult with the encoder that measured the relative linear position. Thereby, the digital gauge was added to know the true linear position so that the strain of the specimen could be computed. The base of the QD holding the modules had a width of 7 cm and a length of 42 cm.

### 2.2. Quantifying Methods for Soft Tissue Material Properties Using a Strain vs. Stress Curve

**Figure 2:**
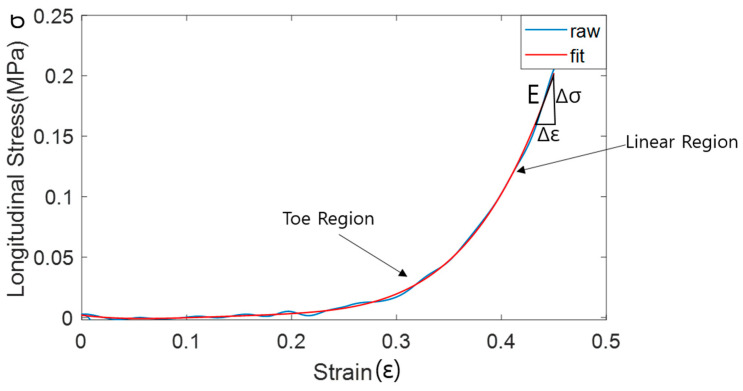
An example of the raw and fitted strain vs stress curve in the longitudinal direction.

Using the QD, tensile force and elongation data can be obtained. The QD can control the maximum elongation length as well as the elongation velocity. Furthermore, repeatable elongation can be possible so that multiple strain and stress curves can be obtained. Based on the known initial dimension of the tissue sample as a length (l), width (w), and depth (d), stress (σ) was computed as force (F) measured from the force sensor divided by area (A), and strain (ε) was computed as length measured from the digital gage divided by the initial length of the tissue sample. Based on the strain and stress curve obtained from the cyclic testing with various testing speeds, young’s modulus of the material at various speeds can be quantified using the QD. Each empirical strain vs stress hysteresis curve from the cyclic testing was resampled for averaging purposes and time normalized to divide the cyclic data into the ascending limb and descending limb of the hysteresis loop. The resampled data were further fitted using the 5th order polynomial function. Then, the young’s modulus (E) can be quantified as Δσ/Δε from the linear region of the ascending limb of the graph, similar to previous researches in computing Young’s Modulus in the biological tissue [1] (Figure 2). 

### 2.3. Quantification of Young’s Modulus of Soft Tissues Using a Cadaver

**Figure 3:**
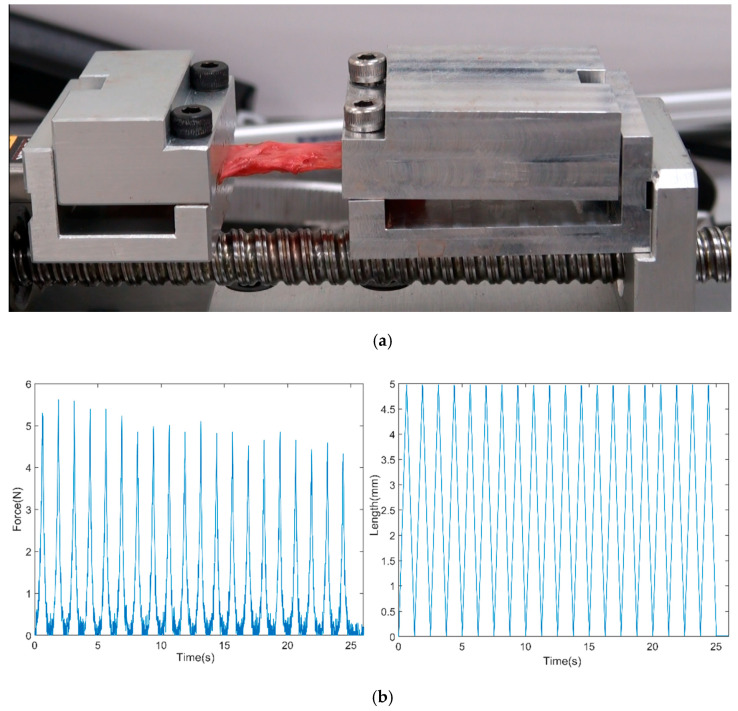
(**a**) An example of the test sample (Quadratus lumborum) during the test (**b**) an example of the force and length raw data from the QD.

One fresh frozen male cadaver was donated from the surgical anatomy education center at Yonsei university health system. The experiment was conducted at the surgical anatomy education center at Yonsei University Health System. Prior to the experiment, the frozen cadaver was thawed and a skilled surgeon prepared the test specimens from the erector spinae muscle (ESM), quadratus lumborum muscle (QL), psoas major muscle (PM), anterior longitudinal ligament (ALL), and posterior longitudinal ligament (PLL) from the lumbar spine. Considering to obtain young’s modulus of the sample in the longitudinal and transverse direction of the fiber, a total of ten test samples were prepared (five samples per direction). Each dimension of the test sample can be found in Table 1. Each test sample was secured on the holding module as Figure 3a and the initial length was adjusted using the manual maneuver not to be slacked between the two brackets. Each test sample was conditioned by a few repeatable cyclic stretches, then applied for 20 repeatable cyclic 5 mm stretch-and-relax trials at 2, 3 and 5 mm/s. Five samples were stretched along with the fiber and five samples were stretched in the perpendicular direction of the fiber. The representative raw data obtained from the device was shown in Figure 3b. The force and length data were collected through Laview at 25 Hz.

### 2.4. Feasibility Test

**Figure 4:**
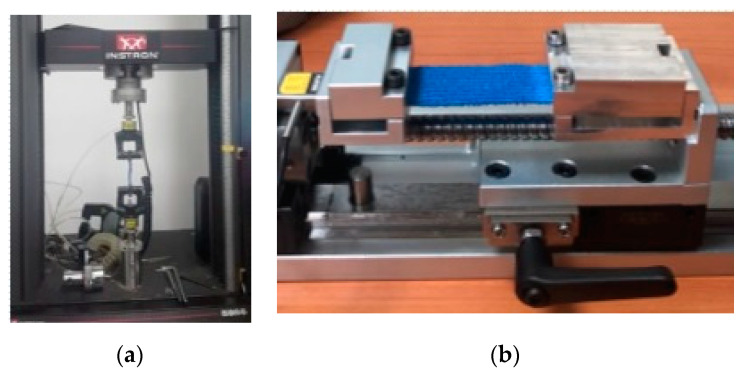
The test setup from (**a**) Instron, and (**b**) the Quantifying device (QD).

A feasibility test was performed using the QD. Young’s modulus (E) of the test material was compared between the value obtained from strength testing machine Instron 5966 and the value measured from the QD (Figure 4). The test material was a piece of the latex-free non-woven cohesive flexible bandage (SPICA) used to secure dressings and other devices. It was chosen because the young’s modulus was close to biological tissue such as muscle and it had an elastic component and it was easy to cut for making specimens. Two test samples were made using stacks of the cohesive flexible bandage to compare the young’s modulus between the two conditions. The first specimen in the longitudinal direction was fixed in the mechanical jaw holders of an Instron 5966 system (Instron, Norwood, MA, USA). Because the mechanical jaw holders could not hold for the width of the 25 mm flexible bandage, only the longitudinal direction of data was collected. 

### 2.5. Statistics

The normality of the data was checked. Most conditions showed normal distribution; thereby, two-way repeated analysis of variance (ANOVA) tests were performed to investigate the effect of speed and direction on each specimen’s Young’s modulus (E). Independent variables were speed and direction, and dependent variables were Young’s modulus (E) of the ESM, QL, PM, ALL, and PLL. If the sphericity assumption is rejected, Greenhouse-Geisser correction was used. If there is a significant effect of speed, direction, or interaction of speed and direction, pair-wise posthoc analysis was performed with Bonferroni corrections.

## 3. Results

### 3.1. Controlling the Speeds of the Quantifying Devices and Allowing a Different Initial Length Setup

The QD was able to control the speeds of the motor so that the specimen can move at a slow speed so that the effects of speeds on soft tissue material properties can be investigated. In our feasibility study, 2 mm/s, 3 mm/s, and 5 mm/s speeds were used for specimen conditioning and elongation. To obtain the stress and strain curve of each specimen from the cadaver, the different initial length was set-up by moving the guide of the handle so that the different length of each specimen from the cadaver can be tested (Table 1). Thus, our developed QD was able to allow a different initial length setup for various specimen samples.

### 3.2. Strain and Stress Curve of the Specimens from the Cadaver and Young’s Modulus of the Materials

**Figure 5:**
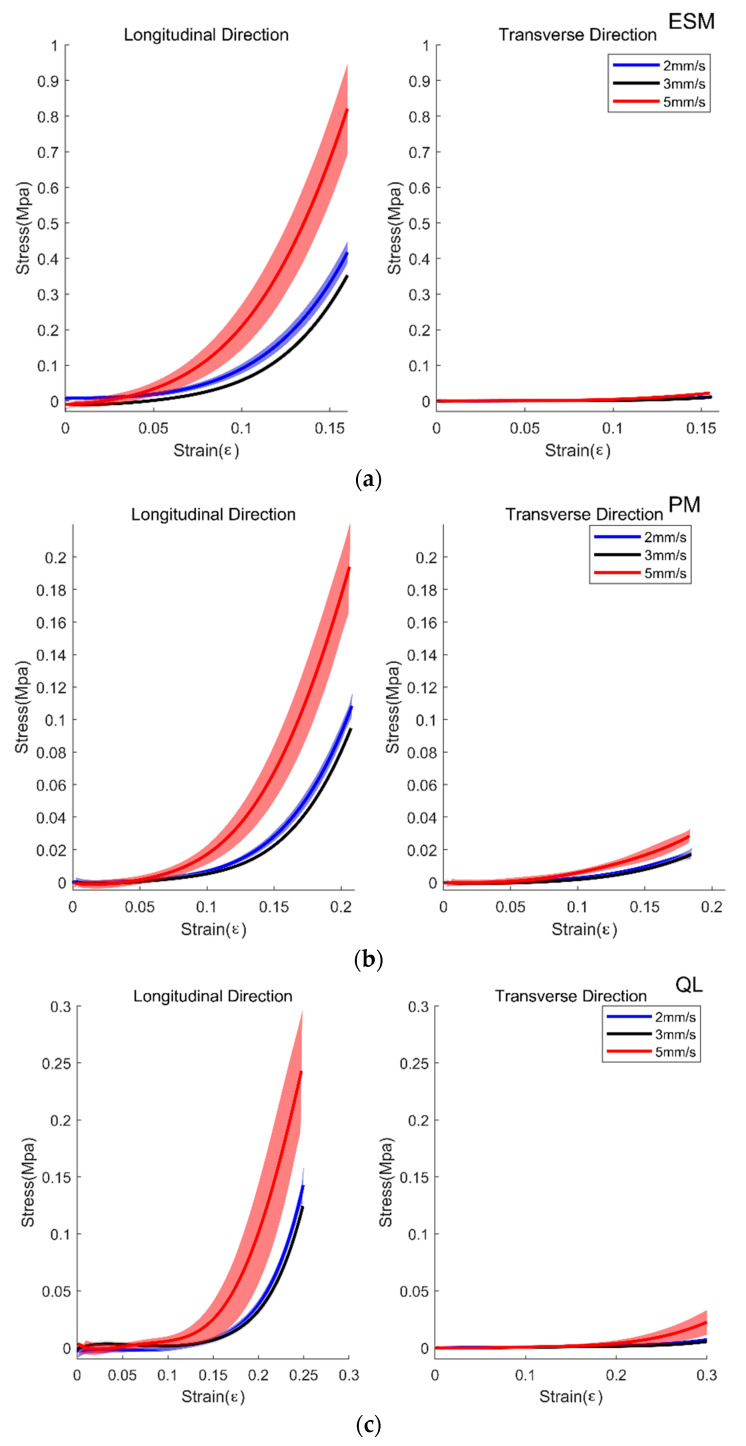
Average Strain and Stress curve of Erector Spinae Muscle (ESM, (**a**)), Psoas Major (PM, (**b**)), and Quadratus Lumborum (QL, (**c**)). The blue line with shading, mean±1SD for 2 mm/s trials; black line with shading, mean±1SD for 3mm/s trials; red line with shading, mean ±1 SD for 5 mm/s trials.

**Figure 6:**
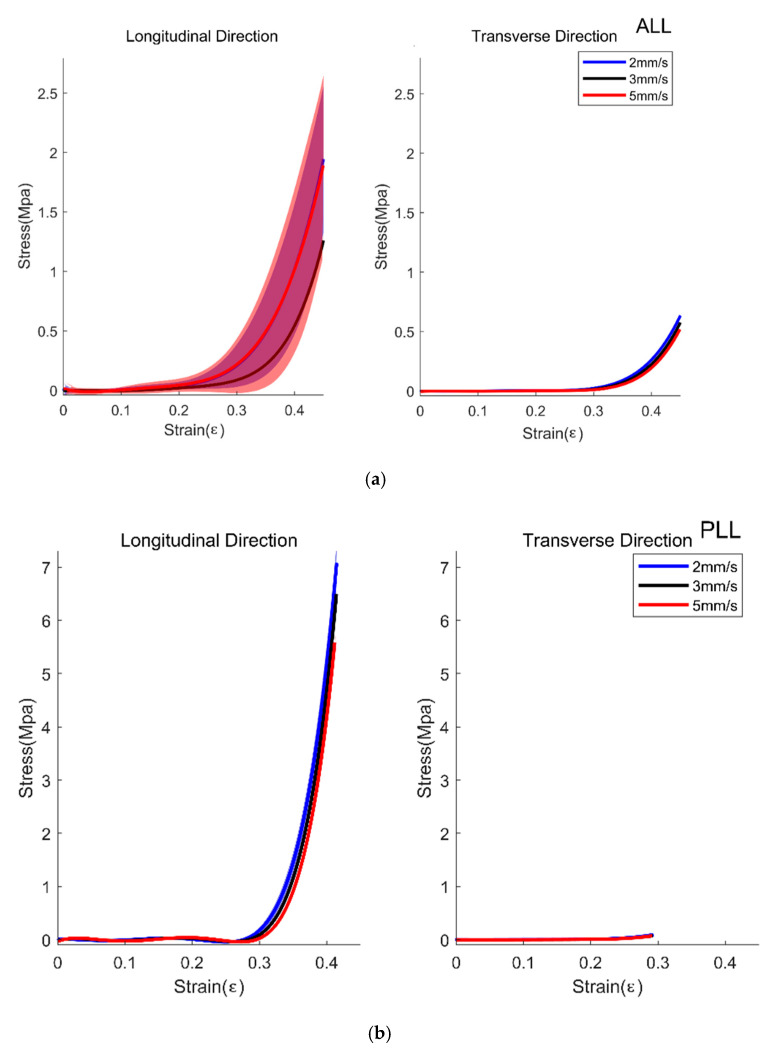
Average Strain and Stress curve of Anterior Longitudinal Ligament (ALL, (**a**)) and Posterior Longitudinal Ligament (PLL, (**b**)). The blue line with shading, mean±1SD for 2 mm/s trials; black line with shading, mean ±1 SD for 3 mm/s trials; red line with shading, mean ±1 SD for 5 mm/s trials.

There was a significant effect of the direction on the young’s modulus of all samples, namely, ESM, QL, PM, ALL, and PLL (*p* < 0.001 for all cases). The young’s modulus of all samples was large in the longitudinal direction compared to the transverse direction as seen from Figure 5 and Figure 6. Velocity-dependent behaviors of the stress and strain were found in the specimens (Figure 5 and Figure 6). There was a significant effect of the speed on the ESM, QL, PM, and PLL (*p* < 0.001, *p* = 0.025, *p* < 0.001, and *p* < 0.001, respectively), and there is a marginally significant effect of the speed on the ALL (*p* = 0.051). Especially, the speed-dependent differences were large in the specimens elongated in the longitudinal direction. Table 2 lists the means and SD of the young’s modulus of ESM, PM, QL, ALL, and PLL in the longitudinal direction and the transverse direction. There was a significant effect of the interaction between the direction and the speed on the ESM, PM, and PLL (*p* < 0.001, *p* = 0.001, and *p* < 0.001, respectively). Posthoc analysis revealed that there were significant differences in the direction between the all samples as *p* < 0.001, and in the speed between the 2 mm/s and the 5 mm/s conditions, and the 3 mm/s and the 5 mm/s conditions of the ESM (*p* < 0.001 and *p* = 0.001, respectively), between the 2 mm/s and the 5 mm/s conditions and the 3 mm/s and the 5 mm/s conditions of the PM (*p* < 0.001 and *p* < 0.001, respectively), and between the 2 mm/s and the 3 mm/s conditions of the PLL (*p* = 0.002), and the 2 mm/s and the 5 mm/s conditions of the PLL (*p* < 0.001), and the 3 mm/s and the 5 mm/s of the PLL (*p* < 0.001).

Within the longitudinal direction of the samples, there were significant effects on Young’s modulus in the ESM between the 2 mm/s and 5 mm/s condition (*p* < 0.001), and between the 3 mm/s and 5 mm/s condition (*p* < 0.001), in the PM between the 2 mm/s and 5 mm/s (*p* = 0.002), and the 3 mm/s and 5 mm/s condition (*p* < 0.001), in the ALL between the 2 mm/s and 3 mm/s condition (*p* = 0.002), and in the PLL between the 2 mm/s and 3 mm/s condition (*p* < 0.001), between the 2 mm/s and 5 mm/s condition (*p* < 0.001), and between the 3 mm/s and 5 mm/s condition (*p* < 0.001).

Within the transverse direction of the samples, there were significant effects on Young’s modulus in the ESM between the 2 mm/s and 5 mm/s condition (*p* = 0.003), in the QL between the 2 mm/s and 5 mm/s condition (*p* = 0.001), and between the 3 mm/s and 5 mm/s condition (*p* = 0.001), in the PM between the 2 mm/s and 5 mm/s condition (*p* = 0.014), and in the ALL between the 2 mm/s and 3 mm/s condition (*p* = 0.008), the 2 mm/s and 5 mm/s condition (*p* < 0.001), and 3 mm/s and 5 mm/s condition (*p* < 0.001).

### 3.3. Feasibility Test

**Figure 7:**
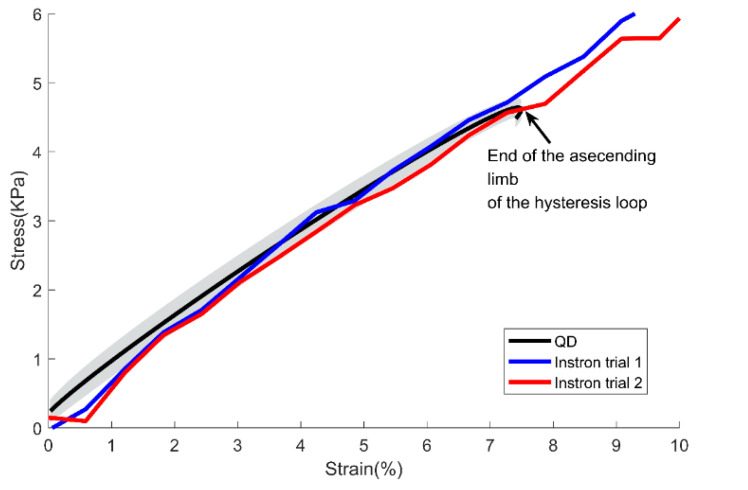
Mean ascending limb of the stress and strain curve from the hysteresis loop obtained from the QD. The black bold line indicates the mean value and the shade indicates ±1 Confidence Interval of the data obtained from the QD. The blue line and the red line indicate each trial from the Instron equipment.

As seen from Figure 7, the slopes of elongation trials from Instron were mainly within the range of confidence interval of the slopes obtained from the QD. The young’s modulus of the Instron trial 1 was 569.9 kPa and the Instron trial 2 was 578.4 kPa, which were within the 95% confidence interval value obtained from QD (the lower bound: 560.3 kPa and the upper bound: 603.0 kPa). While the QD can repeat the stretch-relax of the specimen, the Instron setup used in this study can only stretch until the specimen is reached to failure. Thereby, in Figure 7, the range of strain in the Instron trial exceeds the range of strain obtained from the QD as indicated from the end of the ascending limb of the hysteresis. 

## 4. Discussion

A QD was developed to quantify the anisotropic and viscoelastic properties of musculoskeletal soft tissues. The device can (1) change the stretching speed and adjust the initial length, (2) measure the stretching force and displacement of the specimen in real-time, and (3) provide the young’s modulus of the specimen. In addition to developing the QD, the feasibility of the device was tested using the ten specimens of lumbar spine soft tissues from a cadaver, and the values in the cohesive flexible bandage sample were tested from the Instron and the QD were also compared.

Our uniqueness of the device and study is that the speed of the stress and relaxation was controlled and the young’s modulus can be measured in the longitudinal and transverse direction in both ligaments and muscles for the first time. Furthermore, based on our study, the stress and strain curve of the sample obtained from our developed QD and that from Instron one of the widely used commercial material testing devices were comparable. Compared to Instron, our device was more flexible to adjust the length of holding the initial specimen so that not only the longitudinal direction but also the transverse direction of the sample can be tested for obtaining Young’s modulus. Furthermore, the cost of QD is 1/10 of Instron, which can be further reduced, and also it is more portable compared to Instron. Therefore, our device can be used in a surgical room or various lab environments for testing material properties of soft tissues on the fly when surgery is going on.

In our study, the stretching direction and speed influence the young’s modulus of most test specimens around the lumbar spines indicating the biological tissues had anisotropic and viscoelastic properties. It has been reported that the muscles and ligaments demonstrated the behaviors of transversely isotropic so that the larger stress would show in the longitudinal direction compared to the transverse direction [1,11,12]. In our feasibility study, all samples showed such behaviors. As the previous study reported the young’s modulus of ALL and PLL in the longitudinal direction from the lumbar spine was 20 MPa for ALL and 70 MPa for PLL [13] with considering the subject-variation can be measured up to 64 MPa that can be seen from [14], our results are acceptable and reasonable.

Various methods have been incorporated to investigate muscle material properties and demonstrated inconclusive results. Most recently, shear wave elastography has been used to quantify muscle material properties; however, it has been debating whether the values are the same as young’s modulus that classically defined [15,16]. In the shear wave elastography study, the ESM was in the range of approximately 14-18 kPa [17,18,19]. While there is a lack of information on muscle young’s modulus around the lumbar spine similar to measured from our study, there were few studies that reported muscle young’s modulus with and without aponeurosis. In one study, the large value of young’s modulus of extensor digitorum longus without aponeurosis from the rabbit was approximately 446 kPa in the longitudinal direction and 22 kPa in the transverse direction [11]. In the study investigating the young’s modulus of skeletal muscle cell that originated from normal adult C3H mouse leg muscles, 100–700 kPa range was reported [20]. Compared to those previous studies, our young’s modulus of muscle seemed too high. However, caution might be necessary for the interpretation of results because experimental conditions, types, and the dimension of the specimens, the velocity of elongation, and the presence of aponeurosis can have impacts on the results. Indeed, in a previous study investigating mechanical properties of triceps surae aponeurosis from cadavers, around 200 MPa in the longitudinal direction and around 0.8 MPa in the transverse direction was found in young’s modulus of medial and lateral gastrocnemius and soleus aponeurosis [21]. The aponeurosis is a structure transmitting force generated from a muscle to a tendon, which often includes investigating muscle mechanical properties since it would be difficult to isolate for material testing [11]. In our study, it was challenging to make two samples per location due to the narrow thickness of the muscles and ligaments around the lumbar spine. Thereby, not only muscle tissue but the connective tissue around the muscles including the aponeurosis and fascia can influence to have higher young’s modulus than either isolated muscles without aponeurosis and muscle cells. However, this is a more realistic and practical situation that can be faced during surgery and developing an anatomically realistic biomechanical model. Thereby, a more wide range of material properties in both muscles and ligaments and conditions may need to be considered.

Quantifying the anisotropic and viscoelastic properties of skeletal soft tissues can facilitate the process of identifying pathological conditions of muscles, ligaments, and tendons such as high stiffness of muscles might be due to spasticity and contracture of the muscles, or due to abnormal development because of cancers, aging, or other pathologies. Furthermore, recent trends of developing image-guided spine surgical procedures utilize a biomechanical model to develop a minimum incision and minimum exposure to spine surgery [22]. In that case, knowing the anisotropic and viscoelastic properties of skeletal soft tissues can help to more accurately co-register the location of the spine. While our study only investigated ten samples from a cadaver, investigating more samples from a different type of tissue such as age, gender, and pathological conditions can understand material properties of soft tissues and develop a more accurate biomechanical model.

## Figures and Tables

**Table 1:** Sample dimension. L denotes length, W denotes width, and T denotes a thickness.

	Direction					
Specimen	Longitudinal (mm)			Transverse (mm)		
	L	W	T	L	W	T
Erector Spinae Muscle (ESM)	30	3	4	32	20	8
Quadratus Lumborum (QL)	20	3	8	12	15	6
Psoas Major (PM)	24	9	5	27	16	4
Anterior Longitudinal Ligament (ALL)	11	2	2	11	8	4
Posterior Longitudinal Ligament (PLL)	12	7	3	17	9	4

**Table 2:** Mean (std) of Young’s Modulus of each specimen.

Specimen	2 mm/s	3 mm/s	5 mm/s
Longitudinal Direction (Mpa)			
Erector Spinae Muscle	10.57 (1.52)	10.00 (1.64)	15.84 (2.71) *,+
Quadratus Lumborum	3.45 (0.61)	2.93 (0.83)	3.72 (0.89)
Psoas Major	2.19 (0.26)	2.04 (0.32)	2.87 (0.59) *,+
Anterior Longitudinal Ligament	23.83 (4.95)	20.28 (2.78) *	21.66 (5.17)
Posterior Longitudinal Ligament	141.94 (2.68)	138.23 (2.27) *	126.28 (2.57) *,+
Transverse Direction (Mpa)			
Erector Spinae Muscle	0.32 (0.12)	0.33 (0.14)	0.48 (0.20) *
Quadratus Lumborum	0.61 (0.07)	0.56 (0.11)	0.93(0.19) *,+
Psoas Major	0.32 (0.22)	0.40 (0.26)	0.55 (0.30) *
Anterior Longitudinal Ligament	10.46 (0.34)	9.95 (0.34) *	9.06 (0.42) *,+
Posterior Longitudinal Ligament	2.10 (0.25)	1.78 (0.45)	1.88 (0.47)

* indicates *p* < 0.05 between 2 mm/s and 3 mm/s or 2 mm/s and 5 mm/s, + indicates *p* < 0.05 between 3 mm/s and 5 mm/s.

## Data Availability

The data presented in this study are available on request from the corresponding author.
